# A simple approach for preparation of affinity matrices: Simultaneous purification and reversible immobilization of a streptavidin mutein to agarose matrix

**DOI:** 10.1038/srep42849

**Published:** 2017-02-21

**Authors:** Sau-Ching Wu, Chris Wang, Dave Hansen, Sui-Lam Wong

**Affiliations:** 1 Department of Biological Sciences, University of Calgary, 2500 University Dr., N.W. Calgary, Alberta, T2N 1N4, Canada

## Abstract

SAVSBPM18 is an engineered streptavidin for affinity purification of both biotinylated biomolecules and recombinant proteins tagged with streptavidin binding peptide (SBP) tags. To develop a user-friendly approach for the preparation of the SAVSBPM18-based affinity matrices, a designer fusion protein containing SAVSBPM18 and a galactose binding domain was engineered. The galactose binding domain derived from the earthworm lectin EW29 was genetically modified to eliminate a proteolytic cleavage site located at the beginning of the domain. This domain was fused to the C-terminal end of SAVSBPM18. It allows the SAVSBPM18 fusions to bind reversibly to agarose and can serve as an affinity handle for purification of the fusion. Fluorescently labeled SAVSBPM18 fusions were found to be stably immobilized on Sepharose 6B-CL. The enhanced immobilization capability of the fusion to the agarose beads results from the avidity effect mediated by the tetrameric nature of SAVSBPM18. This approach allows the consolidation of purification and immobilization of SAVSBPM18 fusions to Sepharose 6B-CL in one step for affinity matrix preparation. The resulting affinity matrix has been successfully applied to purify both SBP tagged β-lactamase and biotinylated proteins. No significant reduction in binding capacity of the column was observed for at least six months.

Streptavidin is a tetrameric biotin binding protein. Since its interaction with biotin represents one of the strongest known non-covalent interactions (K_d_ = ~4 × 10^−14^ M), streptavidin-biotin technology has been widely applied for many affinity capture and immobilization applications^1^,^2^. To further extend the applications of the streptavidin-biotin technology, various streptavidin muteins with higher or lower biotin binding affinities have been developed^3^,^4^,^5^,^6^. Engineered streptavidin with reduced biotin binding affinity has gained most of the attention since these engineered proteins have the potential to develop affinity matrices for purification of biotinylated biomolecules in high purity with excellent yield. With the recent advancement in BioID technology^7^,^8^, a proximity dependent biotinylation approach to identify interacting biomolecules, the need to develop engineered streptavidin affinity matrices with reversible biotin binding capability is even stronger. The combination of BioID technology and these matrices potentially allows the capture and purification of interacting molecules. Furthermore, development of reusable biosensor chips and bioreactors is another attractive application of these engineered muteins. Strategies to develop these muteins include replacement of key biotin binding residues in the biotin binding pocket^9^,^10^,^11^,^12^,^13^, deletion of the lid (loop 3–4, a major biotin binding loop) in the biotin binding pocket via the construction of circular permutated streptavidin^14^, development of monomeric streptavidin through the generation of interface mutants^15^,^16^ or hybrid streptavidin^6^,^17^ and creation of a mobile biotin exit door (by replacement of loop 7–8, a rigid loop, with an engineered mobile loop) in the biotin binding pocket to allow the escape of the bound biotin^18^. Another approach to allow reversible interactions between streptavidin and recombinant proteins is the development of streptavidin binding peptide tags with the streptavidin binding affinities ranging from μM to nM. These tags include strep-tags I, II and III^19^,^20^, nano-tag^21^ and streptavidin binding peptide (SBP) tag^22^,^23^. These tags bind directly to streptavidin without the need of biotinylation. Although both the nano-tag and SBP tag bind to streptavidin with comparable binding affinity in the nanomolar range, nano-tag is functional only at the N-terminus of the tagged proteins^21^. In contrast, SBP tag offers greater flexibility and can be inserted to a recombinant protein at N-terminal, internal or C-terminal position, without affecting its function^24^,^25^,^26^. SBP tagged recombinant proteins can be easily affinity purified to high purity in one step using biotin as an eluent^22^,^23^. However, the extremely tight binding between streptavidin and biotin poses a limitation to this technology. As the streptavidin affinity matrix can be poisoned by biotin, it can be used only once in the purification of SBP tagged proteins. With the recent development of a designer streptavidin, SAVSBPM18, which can bind both biotin and SBP tag with the binding affinity in the range of 10^−8^ M, the SAVSBPM18 affinity matrix can be applied to purify biotinylated biomolecules and SBP tagged recombinant proteins in a reusable manner^27^.

To prepare a protein based affinity matrix (e.g. SAVSBPM18 Affi-gel), the standard approach is to chemically immobilize proteins via amino or other functional groups to the activated matrix. Since there are many functional groups on a protein surface, orientation of the chemically immobilized proteins will be fairly random and cannot be fixed. In some cases, the binding pockets may be located very close to the matrix surface and are not available for interaction with the binding partners. Furthermore, some of the key binding residues in the binding pocket of these proteins can react with the activated groups on the matrix. Consequently, these binding pockets are no longer active for binding because of steric hindrance. This lack of orientation-specific immobilization can substantially limit the binding capacity of the resulting matrix^28^. An additional disadvantage of chemical coupling is that this procedure requires the ligand proteins to be purified before coupling. These pure proteins have to be highly concentrated (10 mg/ml or more) for efficient coupling^29^. At these concentrations, proteins with limited solubility tend to aggregate. Furthermore, conditions of the coupling process can be finicky, demanding different activated matrices depending on the nature of the proteins (acidic or basic). It would be ideal to have a simple, efficient and cost-effective approach that combines purification and immobilization in one step to generate the affinity matrix.

Since commercially available agarose based Sepharose matrices are well established as the matrices for affinity chromatography, it is attractive to create a protein fusion that is composed of the protein of interest fused to an agarose binding domain (ABD). Application of the fusion protein to a Sepharose column can potentially affinity purify and immobilize the protein of interest to generate the affinity matrix.

Agarose is a linear polysaccharide containing an average of 400 agarobiose repeating units, a disaccharide with alternating D-galactose and 3,6-anhydro-L-galactopyranose^30^. These polysaccharide chains intertwine to form a porous three-dimensional network in Sepharose beads^31^. Since agarose has many galactose residues, a galactose binding domain can serve as an ABD. An ideal ABD should have the following features. 1. It should be small in size. 2. It should be capable of folding independently in a redox independent manner. 3. It should be galactose/agarose specific with appropriate binding affinity. Although tight binding would be desirable for immobilization, reversible binding can also be an attractive feature since the domain can then serve as a handle for affinity purification of the fusion proteins^32^. 4. Its three-dimensional structure should be well characterized. 5. It should be able to bind to internal galactose residues in the agarose chain. 6. The binding reaction should be metal ion independent so that chelators (e.g. EDTA) can be included in the buffer for protein stabilization. Among the well characterized galactose binding domains available, a C-terminal 14.5-kDa galactose binding domain from the earthworm (*Lumbricus terrestris*) galactose binding lectin EW29 meets all the above-mentioned requirements. This domain is monomeric in nature^33^ and adopts a β-trefoil fold that can be subdivided into α, β and γ subdomains^34^. Both the α and γ subdomains can bind galactose, lactose, melibiose and other galactose derivatives (Fig. 1). The α subdomain shows a higher binding affinity (10 μM for lactose) while the binding affinity of the γ subdomain is lower (2.66 mM for lactose)^35^. Since this ABD domain is naturally located at the C-terminal end of EW29 (residues 131 to 260), we introduced this domain to the C-terminal end of SAVSBPM18 to create a SAVSBPM18-ABD fusion (Fig. 2, panels a and c). A flexible linker sequence was inserted in the middle to project SAVSBPM18 from ABD. The recombinant protein can be produced intracellularly in *E. coli* as a stable fusion protein and affinity purified and immobilized to Sepharose 6B-CL (CL for crosslinked agarose beads) to generate the SAVSBPM18-ABD matrix in one step. The affinity column can be applied to purify both SBP tagged protein and biotinylated proteins in a reusable manner.

## Results and Discussion

### Rationale for the genetic modifications of the agarose binding domain

**Figure 1:**
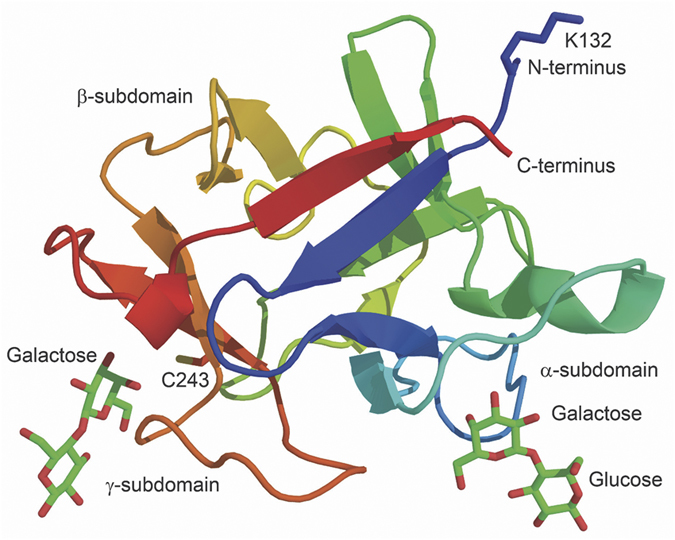
The galactose binding sites in the EW29 galactose binding domain (residue 131 to residue 260 in EW29). Galactose binding sites 1 and 2 correspond to the α- and γ-subdomains, respectively. Each of them is shown to bind to lactose via the galactose residue. The two side chains of residues (K132 and C243) that were selected for replacements are also shown.

Relative to the wild-type galactose binding domain, the galactose binding domain used in this study has two amino-acid substitutions (K132D and C243Q). The natural 29-kDa galactose specific lectin EW29 can be divided into the N-terminal domain and the C-terminal galactose binding domain. Both domains are approximately equal in size. When EW29 was overproduced in *E. coli*, several major degradation products with sizes ranging from 15–17 kDa were observed^33^. Three of these major proteolytic cleavage sites have been mapped immediately upstream of the galactose binding domain, with two of these cleavage sites having the proteolytic cleavage after a lysine. One of these lysine residues, K132, is located within the galactose binding domain (Fig. 1), since residues from P131 to E260 in EW29 are considered to be the galactose binding domain. We introduced a K132D substitution to the synthetic gene encoding this domain to eliminate the possible proteolytic cleavage of this domain from the SAVSBPM18-ABD fusion.

**Figure 2:**
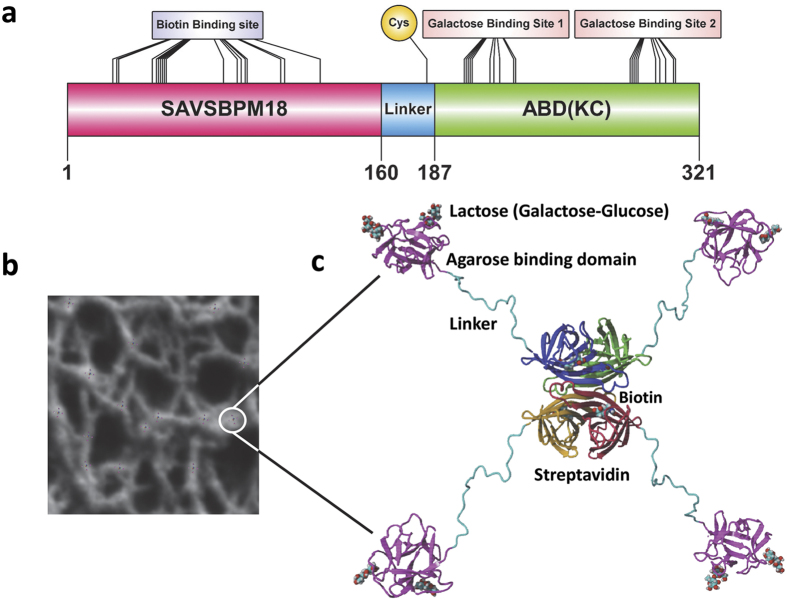
A diagram showing the primary and the modelled structures of the SAVSBPM18-L-ABD(KC) fusion and its immobilization to Sepharose 6B-CL. (**a**) Schematic organization of SAVSBPM18-L-ABD(KC). Biotin binding site in streptavidin, cysteine-183 in the linker region and galactose binding sites in the agarose binding domain are shown. Galactose binding sites 1 and 2 correspond to the α and γ subdomains discussed in the text. (**b**) Binding of SAVSBPM18-L-ABD(KC) to agarose. The structure of SAVSBPM18-L-ABD(KC) is modelled. The diameter of the fusion in this model is 4 nm. In the agarose beads, many agarose strands assemble to form macromolecular strand-like structures with an average diameter in the range of 40–60 nm^31^. Many streptavidin fusions can bind to these structures located on the bead surface. The bound fusions are schematically shown in a larger size (i.e. not in proportion to agarose strands) for the purpose of illustration. One of the bound molecules is marked by a white circle. (**c**) Magnification of a surface bound streptavidin-agarose binding domain fusion. The four streptavidin subunits are colored red, brown, green and blue, respectively. Each subunit has a bound biotin. The agarose binding domains are shown in pink. Each domain interacts with two galactose residues present on the Sepharose bead surface. The galactose in complex with ABD is shown in the form of lactose (Galactose-Glucose). The linker region is shown in cyan.

Although this galactose binding domain has two galactose binding sites (Figs 1 and 2), the binding affinities are relatively weak. It has been shown that the galactose binding domain from the mushroom hemolytic toxin LSLa binds to agarose beads in a dynamic manner^36^. The rapid dissociation of this domain from the beads and its rebinding leads to a fast infiltration of the galactose binding domain from the bead surface to the centre. Structurally speaking, this mushroom toxin derived galactose binding domain also has the β-trefoil fold. The high and low affinity lactose binding sites have affinity around 17 μM and 11 mM, respectively^32^. These values are comparable to those (10 μM and 2.66 mM) from the EW29 galactose binding domain^35^. Hence, the EW29 galactose binding domain may behave in a similar manner. To monitor the dynamic spatial distribution of the EW29 galactose binding domains in agarose beads, a single cysteine (Fig. 2, panel a) was introduced in the linker region just upstream of the galactose binding domain so that a thiol specific fluorescent dye can be coupled to this domain in a site-specific manner. However, since a cysteine (C243) is also naturally located near the γ subdomain for galactose binding, attachment of a bulky fluorescent dye to this cysteine may interfere with galactose binding. Sequence alignment of this galactose binding domain with its homologs indicates that most galactose binding domains have a glutamine in the corresponding position. Therefore, a C243Q mutation was introduced to the sequence encoding this domain, leaving the linker cysteine for coupling to the fluorescent dye. The final genetically modified EW29 galactose binding domain begins with a methionine followed by a 28-residue cysteine containing linker sequence (ASSPGSGPGTAGGPTSGTSPEGDPCTSG) joined to the cysteine-free EW29 galactose binding domain (K132D, C243Q). This domain is designated L-ABD(KC).

### Recombinant production of L-ABD(KC) and SAVSBPM18-L-ABD(KC)

**Figure 3:**
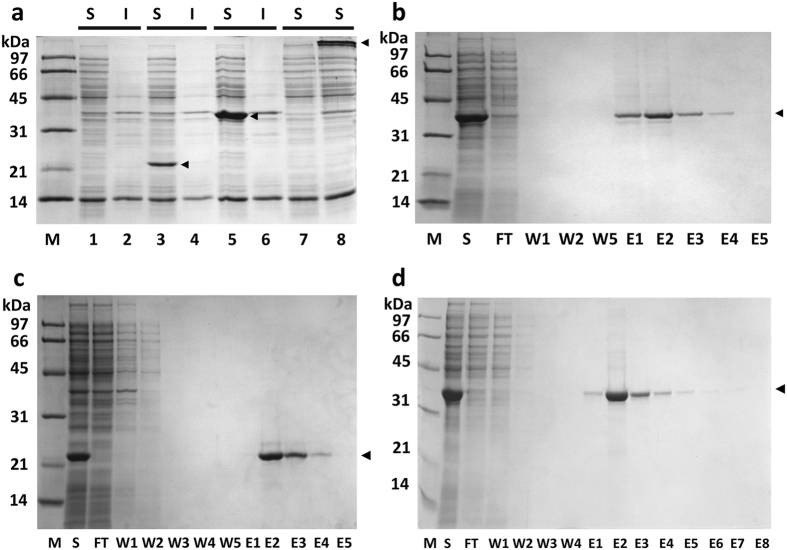
SDS-PAGE analysis of L-ABD(KC) and SAVSBPM18-L-ABD(KC) production and purification. (**a**) Distribution of L-ABD(KC) and SAVSBPM18-L-ABD(KC) in the intracellular fractions of *E. coli*. Cells were disrupted by sonication. Lanes 1 and 2, negative control, *E. coli* BL21(DE3)[pET29B]. Lanes 3 and 4, samples from the L-ABD(KC) production strain. Lanes 5 and 6, samples from the SAVSBPM18-L-ABD(KC) production strain. All samples in lanes 1–6 were boiled before loading onto an SDS-polyacrylamide gel. Lanes 7 and 8, samples were unboiled. Lane 7, negative control; lane 8, SAVSBPM18-L-ABD(KC). Arrowheads mark the positions of L-ABD(KC) and SAVSBPM18-L-ABD(KC). (**b**) Affinity purification of SAVSBPM18-L-ABD(KC) by biotin agarose. Arrowhead marks the position of SAVSBPM18-L-ABD(KC). (**c**) Purification of L-ABD(KC) by Sepharose 6B-CL. Arrowhead marks the position of L-ABD(KC). (**d**) Affinity purification of SAVSBPM18-L-ABD(KC) by Sepharose 6B-CL. Arrowhead marks the position of SAVSBPM18-L-ABD(KC). M, molecular weight marker; S, soluble fraction; I, insoluble fraction; FT, flow-through fraction; W, wash fractions; E, elution fractions.

L-ABD(KC) and SAVSBPM18-L-ABD(KC) are produced intracellularly in *E. coli*. As shown in panel a of Fig. 3, both proteins were expressed predominantly in the soluble form. The observed apparent molecular mass (22 kDa) for the L-ABD(KC) domain is slightly larger than the expected value of 17.5 kDa. This observation is consistent with our previous findings that the presence of a 25-amino-acid linker sequence, which can adopt a random conformation, can cause the apparent molecular mass determined by SDS-PAGE to be ~5 kDa larger than that determined by the mass spectrometry method^37^,^38^. In this case, the linker sequence upstream of the ABD(KC) domain is 28 amino acids in length with somewhat evenly distributed helix breaker residues such as glycine and proline. As well, the monomer of SAVSBPM18-L-ABD(KC) showed an apparent molecular mass of 37 kDa, which again is slightly larger than the expected value of 34 kDa. It is important to note that SAVSBPM18-L-ABD(KC) was stably produced as a fusion protein with no obvious proteolytic cleavage just upstream of ABD(KC) (Fig. 3, panel a, lane 5). Thus, the K132D mutation at the beginning of the ABD(KC) domain does effectively minimize proteolytic degradation at this site.

The production yield for the soluble SAVSBPM18-L-ABD(KC) was estimated to be around 300 mg/liter of cell culture. SAVSBPM18-L-ABD(KC) produced was mainly in the tetrameric state. When the loaded sample was not boiled (Fig. 3, panel a, lane 8), the 37-kDa protein band corresponding to the monomer disappeared with the appearance of an extra protein band with a molecular mass >97 kDa.

SAVSBPM18-L-ABD(KC) readily binds to biotin. When the crude soluble fraction containing the fusion protein was loaded onto a column of biotin agarose in the presence of lactose, SAVSBPM18-L-ABD(KC) was selectively retained on the column and could be eluted by biotin. The fusion protein could be affinity purified in one step using biotin agarose (Fig. 3, panel b).

### Affinity purification of L-ABD(KC) and SAVSBPM18-L-ABD(KC) using Sepharose 6B CL

Both L-ABD(KC) and SAVSBPM18-L-ABD(KC) could be affinity purified to a high degree of purity in one step with crude cell extracts as the starting material using Sepharose 6B-CL (Fig. 3, panels c and d). Sepharose 6B-CL was selected as the matrix in this study because it is relatively uniform in size (diameter in the range of 45–145 μm with the majority around 50 μm) and it is relatively inert with negligible non-specific interactions. In addition, it has many desirable properties including excellent chemical stability under strongly acidic and basic conditions, great tolerance to high flow rate, and broad fractionation range for globular proteins with different molecular weights (1 × 10^4^–4 × 10^6^). As the affinity of the high affinity binding subdomain of this ABD to lactose is in the range of 10 μM, which is not particularly strong, minor leakage of the bound protein during the wash step was occasionally detected with L-ABD(KC). It was more obvious when the wash fractions were concentrated three times or more. In contrast, as SAVSBPM18-L-ABD(KC) has four agarose binding domains per complex (Fig. 2, panel c), it showed stronger binding because of the avidity effect. No leakage was detected with the wash fractions that had been concentrated 10 times. The protein could still be eluted from the matrix within a few fractions (Fig. 3, panel d) without any severe tailing effect.

While Sepharose 6B-CL is our starting matrix of choice, both L-ABD(KC) and SAVSBPM18-L-ABD(KC) have been successfully affinity purified using Sepharose 4B.

### Dynamic spatial distribution of L-ABD(KC) and SAVSBPM18-L-ABD(KC) to agarose beads

**Figure 4:**
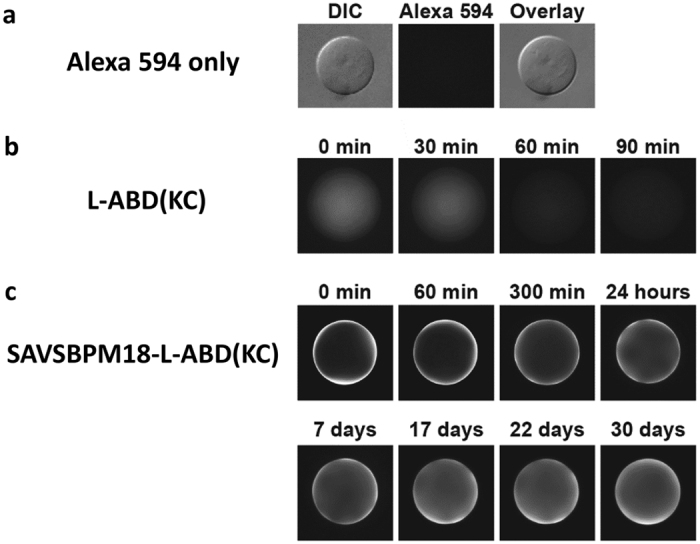
Spatial distribution of monomeric L-ABD(KC) and tetrameric SAVSBPM18-ABD(KC) on agarose beads (Sepharose 6B-CL) at different time points. Time zero is the time point when the beads were mounted for fluorescence microscopy. (**a**) Alexa Fluor 594. The middle panel shows negligible retention of Alexa Fluor 594 dye alone on Sepharose 6B-CL under an epifluorescence microscopy. A differential interference contrast (DIC) filter was applied to visualize the presence of the agarose bead in the left panel. The right panel shows an overlay from both DIC and fluorescence images. (**b**) Alexa Fluor 594 labeled L-ABD(DC) and (**c**) Alexa Fluor 594 labeled SAVSBPM18-L-ABD(KC).

With the purpose of applying L-ABD(KC) for affinity matrix preparation, it is vital to examine how L-ABD(KC) and its tetrameric fusion are distributed in the beads with time. Alexa Fluor 594 which served as the negative control showed no binding affinity to Sepharose 6B-CL (Fig. 4, panel a). In contrast, labeled L-ABD(KC) showed weak binding to the Sepharose 6B-CL beads. Since it took an hour from the time of loading L-ABD(KC) to the column to the time of mounting the beads for fluorescence microscopy (time zero shown in Fig. 4), L-ABD(KC) had infiltrated into the centre of the bead. After 60 minutes, the fluorescence intensity within the bead was significantly reduced (Fig. 4, panel b). Tetrameric SAVSBPM18-L-ABD(KC) showed the strongest binding to the beads. Most of the bound proteins were localized on the bead surface (Fig. 4, panel c). Even after 30 days, most of the bound proteins were still retained on the bead surface, although some degree of infiltration was observed. This observation illustrates that ABD(KC) in the tetrameric state has sufficiently strong avidity to retain the bound proteins on the agarose matrix.

### Application of SAVSBPM18-L-ABD(KC) matrix for affinity purification of proteins

**Figure 5:**
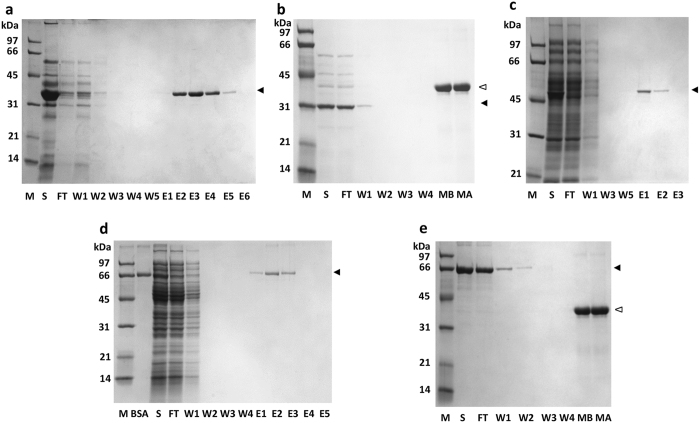
Purification of SBP-tagged or biotinylated proteins using SAVSBPM18-L-ABD(KC) Sepharose column. (**a**) Purification of β-lactamase-SBP from the culture supernatant of *B. subtilis*. Arrowhead marks the position of β-lactamase-SBP. (**b**) Inability of β-lactamase (no SBP tag) from the *B. subtilis* culture supernatant to bind to SAVSBPM18-L-ABD(KC) Sepharose column. MB, boiled matrix (30 μl) from the affinity column before loading of β-lactamase (no SBP tag); MA, boiled matrix (30 μl) from the affinity column after sample loading and washing of the column. Solid arrowhead: β–lactamase; empty arrowhead: SAVSBPM18-L-ABD(KC). (**c**) Purification of biotinylated MBP-AviTag (one biotin per protein) from a crude sample containing intracellular *E. coli* cell extract. Pure biotinylated MBP-AviTag was mixed with the soluble fraction of *E. coli* to constitute the crude sample (S, second lane). Arrowhead marks the position of biotinylated MBP-AviTag. (**d**) Purification of biotinylated BSA (12 biotins per protein) from a crude sample containing intracellular *E. coli* cell extract. Pure biotinylated BSA was mixed with the soluble fraction of *E. coli* to constitute the crude sample (S, third lane). Arrowhead marks the position of biotinylated BSA. (**e**) Inability of non-biotinylated BSA to bind to SAVSBPM18-L-ABD(KC) Sepharose column. MB, boiled matrix (30 μl) from the affinity column before loading of non-biotinylated BSA; MA, boiled matrix (30 μl) from the affinity column after sample loading and washing of the column. Solid and empty arrowheads mark the positions of BSA and SAVSBPM18-L-ABD(KC), respectively. M, molecular weight markers; S, crude sample for all panels except panel e which has the pure non-biotinylated BSA as the sample; FT, flow-through fraction; W, wash fractions; E, elution fractions.

The SAVSBPM18-L-ABD(KC) affinity matrix prepared directly from crude cell extracts of the *E. coli* strain producing the fusion protein was used to purify three different model proteins. For each application, a 1-ml column containing 150 μg bound SAVSBPM18-L-ABD(KC) was used. The first target protein was the recombinant SBP tagged β-lactamase, which was overproduced from *Bacillus subtilis* WB800 via secretion^27^. SBP tagged β–lactamase in the culture supernatant was applied to the affinity column under non-overloading conditions. SAVSBPM18-L-ABD(KC) selectively captured the SBP tagged β-lactamase to the matrix. After washing the column with several volumes of TBS (Tris 50 mM, NaCl 150 mM, pH 7.2), elution with biotin (5 mM) led to the purification of the SBP tagged β-lactamase in one step from the culture supernatant (Fig. 5, panel a). To further demonstrate the specificity of column binding, the culture supernatant of *B. subtilis* WB800 overproducing β-lactamase (no SBP tag)^38^ was applied to the same column and processed using the same scheme. This untagged protein serves as the negative control. After washing with 10 column volumes, a sample (150 μl) of the matrix was boiled to study the bound fraction. Figure 5 (panel b) shows that all β-lactamase was removed in the flow-through and wash fractions. No trace of it was retained on the column.

The second target protein for purification was an enzymatically biotinylated maltose binding protein (MBP-AviTag) mixed with a crude *E. coli* intracellular extract^39^. *E. coli* biotin ligase can enzymatically introduce a biotin to an AviTag^40^. It is of interest to determine whether a recombinant protein with only one biotin per protein can be effectively purified using the SAVSBPM18-ABD(KC) matrix. Figure 5 (panel c) shows that highly purified biotinylated MBP-AviTag was obtained in one step on the affinity column. However, some minor leakage of MBP-AviTag was observed in the wash fractions when a large amount of sample was loaded.

The third target protein was chemically biotinylated bovine serum albumin (BSA) from BioVision. Since this version of BSA has an average of 12 biotins per BSA, it can have a higher binding affinity to the matrix due to the avidity effect. This protein was mixed with crude intracellular extract from *E. coli*, with the amount of biotinylated BSA applied adjusted to a relatively low level with the objective to evaluate the effectiveness of this affinity matrix in purifying a low abundance protein from an intracellular environment. As shown in Fig. 5 (panel d), biotinylated BSA could be affinity purified in one step from the crude extract. No leakage of BSA was observed in the wash fractions even when a larger amount of sample was loaded. Specificity of the column binding was again confirmed using non-biotinylated BSA (Roche) as a negative control (Fig. 5 panel e). Although a large amount of BSA was applied to the column in this control experiment, none of the BSA was found retained on the column as the bound fraction. All BSA was removed in the flow-through and wash fractions.

In all three purification applications, SBP tagged β-lactamase, enzymatically biotinylated MBP-AviTag (one biotin per protein) and chemically biotinylated BSA (12 biotins per protein), high levels of purity were achieved with recovery in the range of at least 85%.

Experiments were conducted to compare the performance of the affinity column generated directly from the crude cell extract containing SAVSBPM18-L-ABD(KC) versus that prepared with SAVSBPM18-L-ABD(KC) previously purified by Sepharose 6B-CL or biotin agarose. Application of the columns on the purification of the chemically biotinylated BSA shows that the column performance is comparable. Thus, it is possible to consolidate the purification and immobilization processes to generate the SAVSBPM18-L-ABD(KC) affinity matrix in one step without compromising the column performance. Generation of the affinity matrix in this way has the added advantages of greater simplicity and cost effectiveness.

### Comparison of performance of SAVSBPM18-L-ABD(KC) matrix with SAVSBPM18 Affi-gel

**Figure 6:**
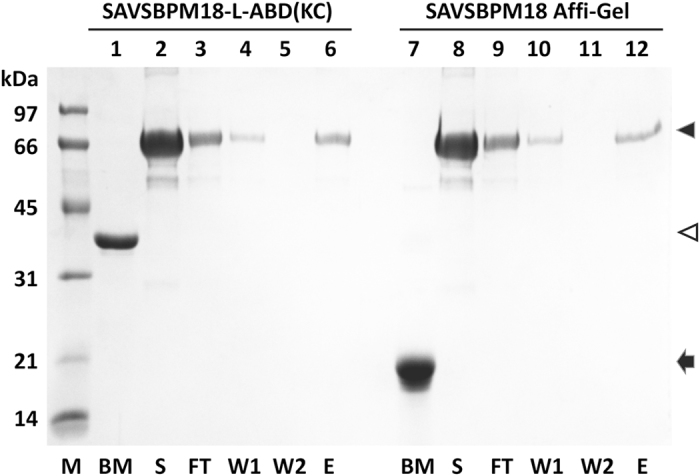
SDS-PAGE analysis comparing performance of SAVSBPM18-L-ABD(KC) matrix with SAVSBPM18 Affi-gel for purification of biotinylated BSA. 1-ml column containing approximately 1 mg of SAVSBPM18 or its fusion was used for each matrix. 1 mg of biotinylated BSA was loaded to each column. Lanes 1–6 are samples from SAVSBPM18-L-ABD(KC) column. Lanes 7–12 are samples from SAVSBPM18 Affi-gel column. M, molecular weight marker; BM, boiled matrix [2 μl for SAVSBPM18-L-ABD(KC); 3 μl for SAVSBPM18 Affi-gel]; S, loaded sample; FT, flow-through fraction; W, Wash fractions; E, concentrated, pooled elution fraction. The volume for each fraction (S, FT, W1, W2 and E) is 1 ml. Volume applied to the gel: 4 μl for S, FT and W; 1 μl for E. Solid and empty arrowheads mark the positions for BSA and SAVSBPM18-L-ABD(KC), respectively. Arrow indicates the position of SAVSBPM18.

The soluble fraction of the *E. coli* strain producing SAVSBPM18-L-ABD(KC) was loaded onto Sepharose 6B-CL to saturate the matrix with the fusion protein. Approximately 1 mg of SAVSBPM18-L-ABD(KC) was captured per ml of Sepharose 6B-CL matrix. In parallel, purified SAVSBPM18 was covalently coupled to activated Affi-Gel 15 (Bio-Rad)^27^. The Affi-gel contained 1,025 μg of SAVSBPM18 per ml of matrix. Both matrices were used to capture biotinylated BSA (BioVision, 12 moles of biotin per mole of BSA), which was applied at an amount that exceeded the binding capacity of the column. Analysis of the distribution of the biotinylated BSA in the flow through, wash and elution fractions showed that at saturation, 1 ml of SAVSBPM18-L-ABD(KC) Sepharose 6B-CL could bind 730 μg of BSA, while 1 ml of SAVSBPM18 Affi-gel could bind 660 μg of the biotinylated protein (Fig. 6). Thus, relative to SAVSBPM18 Affi-gel, the SAVSBPM18-L-ABD(KC) matrix showed good binding capacity in binding biotinylated BSA.

As biotinylated BSA (12 biotins per BSA) binds more strongly to the matrix than biotinylated MBP-AviTag (1 biotin per protein), 10 mM (instead of 5 mM) of biotin was used to elute the biotinylated protein from the column. As well, the column was incubated with the eluent for 30–60 minutes before collecting the eluted fractions. Even with this tight-binding protein, complete elution was accomplished within eight elution fractions (1 column volume per fraction). No remaining biotinylated protein was found on the matrix as analyzed by SDS-PAGE. After each run, the column was regenerated by extensive washing with TBS. The same column was reused five times to capture overloading amounts of biotinylated BSA over a 6-month period. No obvious degeneration in column performance was detected.

### Potential applications

The key success of this approach for preparing an affinity matrix relies on the avidity effect mediated by tetrameric SAVSBPM18-L-ABD(KC). Based on the same principle, affinity matrices involving L-ABD(KC) fusions can also be prepared for different versions of streptavidin/avidin and their derivatives for affinity purification, capture and immunoprecipitation applications^41^. These include wild-type streptavidin and avidin^5^,^6^,^42^, SAVSBPM32^39^, Strep-Tactin^43^, traptavidin^3^ and many other streptavidin/avidin muteins with different biotin binding affinities^4^,^5^,^44^. Besides being used in the traditional column format, an SAVSBPM18-L-ABD(KC) affinity matrix can also be easily prepared in the magnetic bead format with agarose coated magnetic beads for protein purification.

The ABD(KC) fusion technology is not limited to streptavidin/avidin based proteins. Essentially, affinity matrices for any oligomeric proteins with four or more subunits, such as various tetrameric lectins^45^,^46^,^47^ and DNA/RNA binding proteins, can be generated based on the same concept.

Besides purification application, this ABD fusion approach also has many potential applications in nano-technology. Agarose coated porous silicon nanoparticles and agarose based hydrogel beads have been well developed as nano-delivery systems for controlled drug release^48^,^49^. It would be ideal to equip these drug delivery nanoparticles with targeting specificity. If these particles can be loaded with streptavidin-L-ABD(KC) fusions, streptavidin can then be applied to capture biotinylated antibodies that recognize specific cell surface markers. These nanoparticles can become specific drug delivering agents for cancer therapy and other applications. Furthermore, a point-of-care device for rapid and on-site diagnosis of cardiovascular diseases has recently been successfully developed^50^. This device has a micro-sized agarose bead as part of the immune-detection unit. Streptavidin-L-ABD(KC) fusions can be included as bridging molecules to capture biotinylated monoclonal antibodies for diagnostic purpose.

## Methods

### Plasmid construction

The synthetic gene encoding the engineered galactose binding domain from the earthworm (*Lumbricus terrestris*) galactose binding lectin EW29 was obtained from Bio Basic Canada, digested with NdeI/HindIII and inserted into NdeI/HindIII cut pET29B to generate pET29B-L-ABD(KC). This galactose binding domain sequence corresponds to the sequence of residues 131–260 in the EW29 lectin with two changes (K132D and C243Q). A 28-residue cysteine containing sequence was added to the N-terminal end of the galactose binding domain. This flexible sequence serves as a linker (L) for protein fusion construction. To generate pET29B-SAVSBPM18-L-ABD(KC), a synthetic gene encoding SAVSBPM18-L-ABD(KC) was digested with NdeI/HindIII and inserted into NdeI/HindIII cut pET29B. The plasmids were transformed into *E. coli* BL21, which serves as the expression host.

### Production and purification of L-ABD(KC) and SAVSBPM18-L-ABD(KC) for fluorescent microscopy

*E. coli* cells were grown in a shake flask at 30 °C in lysogeny broth (1% tryptone, 0.5% yeast extract, 1% NaCl) containing kanamycin (30 mg/l) until absorbance at 600 nm reached 0.6–0.8. IPTG was added to a final concentration of 0.2 mM to induce protein expression. Growth continued for 3–4 hours at 26 °C. Cells were harvested by centrifugation.

To extract L-ABD(KC) and SAVSBPM18-L-ABD(KC), cell pellets were disrupted by a French pressure cell press (Spectronic Instruments). The soluble fraction was separated from the insoluble fraction by centrifugation (17,000 g, 4 °C, 20 min) and dialyzed against TBS (Tris 50 mM, NaCl 150 mM, pH 7.2). This buffer was used for all column chromatography and was used for both column binding and washing. L-ABD(KC) and SAVSBPM18-L-ABD(KC) in the dialyzed samples were purified by affinity chromatography using Sepharose 6B-CL (GE Healthcare Life Sciences). After sample application, the column was extensively washed with TBS. Bound proteins were eluted with TBS containing 20 mM lactose (Sigma). Fractions of interest were pooled, concentrated and dialyzed against TBS to remove the lactose. Protein concentration was determined using Bio-Rad Protein Assay Dye Reagent.

SAVSBPM18-L-ABD(KC) can also be affinity-purified on a biotin agarose column. The soluble fraction containing the fusion protein was loaded onto a column of biotin agarose (Sigma) that had been equilibrated with TBS containing 20 mM lactose. After extensively washing the column with TBS containing 20 mM lactose, the bound protein was eluted by the same buffer containing 5 mM biotin.

To prepare dye conjugates of L-ABD(KC) and SAVSBPM18-L-ABD(KC) for fluorescence microscopy, Alexa Fluor 594 C5 maleimide (Molecular Probes) was conjugated to the purified proteins according to the manufacturer’s instruction. At the end of the reaction, excess free dyes were removed by extensive dialysis against TBS.

### Microscopy of the distribution of Alexa Fluor 594 labelled L-ABD(KC) and its fusion on Sepharose 6B-CL

Three 150-μl Sepharose 6B-CL columns were packed and loaded with Alexa Fluor 594, Alexa Fluor 594 labelled L-ABD(KC) and Alexa Fluor 594 labelled SAVSBPM18-L-ABD(KC), respectively. To mimic the column washing process, each column was washed with 6 column volumes of TBS. The top layer of the beads was collected and resuspended in 0.25 ml of TBS. Beads taken at each time point were mounted onto slides and examined under Axio Image Z.1 microscope equipped with AxioVision (version 4.9.1.0) software (Zeiss). Images were taken at 1 μm per optical section with constant exposure time.

### Preparation of SAVSBPM18-L-ABD(KC) matrix for affinity purification applications

5 ml soluble fraction of the crude cell extract of *E. coli* strain producing SAVSBPM18-L-ABD(KC) was dialyzed against TBS and loaded onto 10 ml Sepharose 6B-CL (GE Healthcare Life Sciences). The matrix was washed extensively with TBS. The amount of ligand protein loaded under this condition was below the binding capacity of the column since no SAVSBPM18-L-ABD(KC) was observed in the flow-through and wash fractions. The amount of bound SAVSBPM18-L-ABD(KC) was estimated to be approximately 150 μg per ml of matrix. SDS-polyacrylamide gel (SDS-PAGE) analysis of the boiled matrix showed that binding of the proteins was highly specific with over 95% of the bound protein profile composed of SAVSBPM18-L-ABD(KC).

### Other methods

Schematic drawing of SAVSBPM18-L-ABD(KC) with its size proportional to the sequence length is prepared using the Illustrator for Biological Sequences program^51^. The three dimensional model of SAVSBPM18-L-ABD(KC) is prepared using the Yasara Structure package^52^.

## Additional Information

**How to cite this article**: Wu, S.-C. *et al*. A simple approach for preparation of affinity matrices: Simultaneous purification and reversible immobilization of a streptavidin mutein to agarose matrix. *Sci. Rep.* **7**, 42849; doi: 10.1038/srep42849 (2017).

**Publisher's note:** Springer Nature remains neutral with regard to jurisdictional claims in published maps and institutional affiliations.
